# Physiology

**Published:** 1825-01-01

**Authors:** 


VIII.

### Quarterly Periscope

OF

### PRACTICAL MEDICINE;

BEING

### The Spirit of the Medical Journals,

*Foreign and Domestic;*

WITH COMMENTARIES.

I.

### Physiology.

——————— latas à numine leges
Religiosa doce, mentesque cupidine veri
Allice.

1. *Acid in the Human Stomach.** The complaint of *acidity* in the stomach is so common, that it seems wonderful no accurate investigation of the nature of this acidity should have ever taken place. The investigation is not without difficulty, on account of the heterogeneous substances lodged in the stomach previous to vomiting, which rarely allows of a discharge of acid, of a uniform quality, so as to afford an opportunity for a fixed course of analysis. A case, however, fell under our author's observation, where he was enabled to institute a series of experiments upon this obscure subject, and the results are now laid before the profession.

A young woman, named Hutchinson, in the Hospital for Incurables (to which Dr. G. is physician) has been, for several years, subject to fits of vomiting, lasting from ten to fourteen days, and recurring at intervals of about five weeks. These attacks are preceded and accompanied by pain and tenderness in the epigastrium—constant retching succeeds, and almost every thing swallowed is instantly rejected. Her sufferings continue day and night, almost without intermission. Several pints of fluid are daily ejected—sour in smell, and so acrid in nature, as to cause inflammation and excoriation of the throat and other soft parts with which it comes in contact. This phenomenon tempted our author to try whether the fluid contained any free acid. A quantity of what she threw up in the morning was collected, and suffered to stand at rest, when it soon separated into three distinct parts—the uppermost, a red

* Dr. Robert James Graves. Dublin Transactions, Vol. iv.

frothy mucus—the middle, ropy, transparent, and of a straw colour—the lowest, similar, but mixed with a variously coloured and flaky sediment. The middle portion was selected for experiment. It instantly reddened litmus paper, the red colour not destructible by heat. With muriate of barytes it gave no precipitate; but with lime water, a precipitate of a yellowish white colour.

"Oxalate of ammonia gave a white precipitate: that from the nitrate of silver was copious, and, when exposed to the light, assumed a bluish-black colour. The precipitate from acetate of lead was copious, and soluble in dilute nitric acid: its examination with the blow-pipe seemed to prove that it contained both the muriate and phosphate of lead. When the liquor was distilled, no acid came over; what remained in the retort had become more acid, contained a good deal of coagulated matter, and, when the heat was long or continued, it finally became a thick extract, soluble with effervescence, in dilute nitric acid." 319.

These trials shewed that the fluid contained mucus, a free acid not volatile—phosphate of lime, and one or more salts containing muriatic acid. Our author tried whether the fluid contained either urea or uric acid, but careful experiments negatived their presence. The probable presence of lactic acid was now suspected, and our author instituted a series of chemical experiments to ascertain this point. They are detailed at pages 320-1-2 of the work before us, and to these we must refer for particulars. It is sufficient to say that these experiments, in our author's opinion, fully identify this acid with the lactic acid—and that it differs materially from the acetic acid with which it has been often confounded, inasmuch as it may be evaporated to dryness, re-dissolved, and this operation several times repeated, without any considerable loss of acid, while the acetic acid, on the contrary, is extremely volatile.

"As all the fluid vomited by Hutchinson during twelve days possessed the property of instantly and decidedly reddening litmus paper, the quantity of uncombined lactic acid which came away during this period must have been considerable. This long-continued formation of so much acid, where the digestion was as it were completely at a stand, and in a stomach constantly discharging its contents, cannot be accounted for but on the supposition of its being the product of a morbidly-increased secretion.

"As the acid which I examined possesses very considerable chemical powers, being capable of dissolving the oxides of lead, silver, copper, and mercury, while it dissolves metallic iron and zinc with effervescence, a case of its secretion in such large quantities by the human stomach puts us in possession of a new fact which may probably throw light upon the functions and diseases of that important organ." 323.

Berzelius was enabled to detect lactic acid in all the fluids of the human body—and it is only in animal fluids that it is to be found. It cannot be formed by fermentation or any process independent of vitality, agreeing in this respect with urea, uric acids, and other substances occurring only as products of animal secretion.

Since the sensible qualities of the fluid vomited by this patient, and its effects on the throat, mouth, and teeth, were exactly the same as in other cases where the contents of the stomach turn sour previously to being discharged, our author thought it might be reasonably concluded that this acidity is, in general, owing to the presence of *lactic* acid, and he anxiously sought an opportunity of putting this opinion to the test of experiment.

"Such an opportunity soon presented itself in the case of a young gentleman, who, besides the usual symptoms of feverish cold, complained of sickness of the stomach. He vomited during my first visit, and complained much of the sourness of the fluid, which, in taste, he compared to gall. I had some of it brought home, and as it instantly reddened litmus paper, I immediately commenced the process before detailed, and found that the fluid was very strongly impregnated with free lactic acid. As this patient in a few days recovered his usual state of good health, his case abundantly proves, that even a temporary sourness of the stomach may be owing to a morbidly-increased secretion of lactic acid." 324.

Montegre, Dumas, and Philip, have shewn that, during chymifaction, the food acquires a degree of acidity, and is capable of reddening litmus paper. Montegre supposes it to be acetic acid, but acknowledges that its sensible qualities are different, and that it is less volatile than the said acid. M. Montegre has also shewn that what Spalanzani believed to be a peculiar fluid, endued with considerable solvent powers, termed the gastric juice, is merely a mixture of saliva and mucus—"*acid when digested, but not acid when undigested.*" This acid mixture of saliva and mucus has lately been examined by Chevreul, who found that its acidity was owing to lactic acid. "The sourness of the stomach, so much complained of by dyspeptic patients, may, I have no doubt, be referred to the same origin; and this could not fail to prove of considerable practical importance, by teaching the comparative value of the remedies used in that disease, some of which can merely neutralize the secreted acid, while others exert a salutary influence on the secreting surface." The influence of the nervous system being so powerful, it is obvious why sourness of the stomach and acid vomitings, the products of diseased secretion, are so often the almost immediate consequences of mental emotions. It has long been supposed that iron-filings owe their activity to an acid met with in the stomach or bowels. Physicians rightly concluded that the iron must have been converted into a salt, before it could blacken the fæces or affect the constitution, and it is an old observation that this effect takes place most expeditiously in patients suffering under acidity. Luckily, mercury and silver, the salts which are most poisonous, are not acted on by the lactic acid, unless when in the form of oxides. This may serve to account for the escape of persons who have swallowed silver coins or taken metallic mercury. It may also account for some persons being violently purged and griped by the common blue pill, an accident probably owing to its being too rapidly converted into a salt, by meeting with lactic acid in the alimentary canal. "In children there is often a predominance of acid, which renders it prudent to combine an absorbent with the mercurial oxide: chalk has been long used for this purpose, and by guarding against the too rapid formation of a mercurial salt, it serves to render this preparation very mild and safe." We shall be anxious to learn the results of Dr. Prout's researches into the nature of the gastric juice, as laid, we believe, before the Royal Society.

---

2. *Panic Fever.** The influence of mind on matter is now so well known that Dr. Hamilton needed not to repeat the old story of the Prince of Orange curing the Breda Garrison of scurvy, by a few phials of water, introduced as a specific for that disease. He might have quoted, if not "wise saws," at least more "modern instances," illustrative of the panic which spread its baneful influence over the sensitive minds of the Magdalenes of Edinburgh. The fact, however, which is recorded by Dr. Hamilton, is interesting, and we shall present a short account of it to our readers.

On visiting the Magdalen Asylum, on the 2d of April, 1821, our author found that a girl who had gone, in her usual health, to the wash-tub in the morning, was now in bed, having been seized with the usual symptoms of fever. There was an epidemic at the time in the town; and contemplating the possibility of *infection*, Dr. H. had the patient sent to the sick room—her clothes immersed in water—and her room ventilated and locked up. Next day, the fever continuing, she was sent to Queensberry-House, and *fumigation* added to the other prophylactic measures. On the 3d day, two other girls were added to the list, and conveyed to the same destination, the precautionary measures, including the fumigating incantation, being again put carefully in force. We quite agree with Dr. Hamilton that, "the *anxious* employment of these precautions probably attracted the attention and awakened the fears of the inmates, who, cut off from intercourse with the world, are peculiarly alive to all that takes place within the walls of the Institution." A decided alarm now spread among the women, heightened by the idea that the fever had crept into the asylum with the clothes that were sent there to be washed. 4th day, three others had sickened early in the morning, and eight more in a few hours afterwards. All these were sent off to the hospital—all apparently very ill, some with cold tremors, others with flushed face and full pulse, some with nausea, head-ache, vomiting, &c. The linen was drowned, the rooms fumigated, and every thing done to destroy the *contagion* of a few women taken suddenly ill, and where there was no time for the generation of a particle of contagion, in whatever way the fever might have been first introduced. Next day

---

* "The Influence of Panic in propagating Contagious Diseases." By Robert Hamilton, M.D. Surgeon to the Magdalen Asylum of Edinburgh. Ed. Transactions, vol. i. p. 296 *et seq.*

(5th day) our author visited those sent to the hospital. "They all appeared decided cases of idiopathic fever." In the evening of the same day, the panic fever had spread to eight more of the Magdalenes. Thus, in four days, 22 out of a community of 50 individuals became stricken with fever. "The minds of the most stout-hearted in the house, not excepting the (*medical?*) superintendants, participated in the alarm." Dr. Hamilton, however, emancipated himself from the thraldom of fear—went to the sick-room—and, in decided language, pronounced the whole to be a delusion—"that they were yielding to their fears alone, and getting ill merely because others had done so before them." The tide of opinion now turned, and set briskly in an opposite direction. "Of the eight patients then in the sick-room, several recovered in the course of the same night." Next morning some of them took to their employments, and seven out of the eight were all at their usual occupations in a few days. None of the inmates sickened for three weeks after this period, when some of those that were first sent to hospital having returned and relapsed, two or three fresh cases occurred, when all was well again.

We agree with our author in his conclusions; but we think he must acknowledge that, if to him the merit of checking the panic fever belongs—so also does the charge of first contributing to spread it, by the ultra and unnecessary measures put in force to destroy the phantom contagion, existing only in his imagination.

---

3. *Physiological Effects of Lightning.** In the second number of the 10th vol. of Schweigger's Journal, we read of a case which may throw some light upon the manner in which this dreadful meteor acts upon the human frame.

Two cars were going along a hollow road, bounded on each side by a forest. In the first were seated two brothers of the name of Teele, the one, ætat 33, the other, 29. In the second, their nephew, ætat 20, and M. Decker. The lightning struck successively the horse of the first car, the brothers, Decker and his companion, the latter of whom did not survive the accident. The horse became stiff upon the spot; the skin of the belly was torn in all its lower part, the mouth open, and the teeth blackened. The flash struck young Teele as it passed his umbrella, which was thrown, with his watch, 24 feet from the chaise: this was pierced by an opening half a foot in diameter. The body, carried to the next village, was put into a lukewarm bath and rubbed; blood ran from the nose, mouth, and ears, but no sign of life reappeared. The mouth and nose were blackened, the skin of the arms and hands, both of which grasped the stick of the umbrella, was furrowed even to the bone; the sleeves of the coat and shirt were torn; but the lesions of the skin were not at all like the blisters and eschars which follow the application of red-hot iron. The skin seemed to have been

* Gazette de Santé, No. xviii. Juin.

raised by a very rough friction; the clothes shewed no mark of a singe, but were torn as though by the rapid passage of a sharp pointed instrument. Decker, who was in the same car, received, at the same instant, so violent a blow in the lower part of the abdomen, that he was hurled out, and remained senseless for half an hour. When undressed, a deep redness, without any open wound, was found in the place where he felt the stroke. He was able to resume his route immediately.

The two brothers were much injured by the lightning, but however they soon recovered themselves. We may follow the track of the electric spark, and observe the nature of the wounds caused by it. As they were sitting beside one another when struck, the spark first touched the head of the eldest, tore into several pieces the velvet cap which he wore, grazed the temporal bone an inch above the left ear, then went behind it; slightly scorching the skin it descended upon the neck, traversed the nape obliquely, and mounted again towards the right ear: there it scratched the interior of the ear near the tragus and the antihelix, then fell upon the right shoulder, passed under the chin, over the right breast, upon the arm, and, returning over the back, descended the length of the vertebral column to the sacrum.

In this last part of the course of the lightning the skin was not cut, but only a little raised and very red. Marks of the same kind were seen across the arms, which, with the tearing of the clothes, shewed the zig-zag direction of the spark, passing alternately from the right side of the younger brother to the left of the elder. It had fixed upon the former, where it met with some pieces of metal which were in his waistcoat pocket; there it had raised the skin for a space the size of the hand; then descending the left side of the region of the pubis, it had traversed the interior surface of the thigh, the ham, and calf of the leg. A steel chain which the younger brother carried in his fob had conducted the spark to the groin, where a part, the size of the chain, had been stripped of skin and marked with a deep wound. The breadth of the marks was in general about two inches. The wounds were most extensive and deepest at the intersections of this track; many were very painful and suppurated abundantly. The skin had been rolled into dense folds on the right and left by the rapid passage of the electric fluid, the wounds were not bloody, nothing indicated a lesion of organs by fire or heat, but the effect might be compared to the grazing of a ball on the surface of a limb.

The brothers having recovered their senses, experienced violent nausea, and vomited after a few cups of tea; at first some blood came away: notwithstanding the extent of the injury they had no fever. The eldest was quite deaf on the day of the accident, the next day hearing was somewhat recovered, there remained some paralysis in the stricken limbs: the wounds cicatrized.

A year after, (the accident happened in May, 1821) the elder brother was still a little deaf, with a disposition to drowsiness. The younger had had an inflammatory fever and was subject to a periodical weakness before unknown. Their nervous system had suffered much

4. *Spinal Marrow and Nerves.* Professor Bellingeri has recently published some anatomico-physiological remarks on the medulla spinalis and its nerves, containing many facts, and some curious speculations. He has endeavoured to ascertain the relative distribution of the grey and white substances in the spinal marrow, by hardening the chord in nitric acid much diluted with water. The professor comes to the conclusion, that the spinal chord is composed of two substances—grey and white—the grey in the centre, the white on the surface—that its structure is fibrous—that the quantum of white substance in the whole spinal marrow, is much greater than of grey; except in the sacral region, where the proportions are either equal or in favour of the grey. The form of the latter resembles the figure of an X. The anterior horns of the grey matter no where reach so far as the periphery of the chord. Considerable difference in the distribution of these two substances in man, as compared with other animals, is observable; and this phenomenon is made use of by our author in explanation of certain functions which shall be immediately alluded to. From the arrangement of the grey and white matter in man and in animals, Bellingeri concludes that the spinal marrow is divided into six fasciculi, of which, two are anterior, two lateral, and two posterior. The anterior fasciculi are nearly, but not entirely, divided from each other—in the same way they are separated from the lateral;—but the posterior are entirely detached from each other and from the lateral. This arrangement holds good both in man and in animals. But the proportions vary considerably. Thus, the posterior fasciculi in man, (excepting in the lumbar and sacral regions) are thicker than the anterior; whereas in the ox, kid, and birds, the anterior fasciculi are rather thicker than the posterior, except in the superior cervical region in the ox, and in the inferior lumbar and sacral regions in the ox, kid, and birds. Our author agrees with Soemmering that the spinal chord is chiefly composed of prolongations from the cerebellum—or, at all events, communicates chiefly with it. It is demonstrated, he avers, that the anterior roots of the spinal nerves have a triple origin—first, from the anterior fasciculi—secondly, from the region of the anterior lateral fissures—and lastly, from the lateral fasciculi. There are, however, some fibres which rise directly from the white substance of the anterior and lateral fasciculi—and some that appear to come from various parts, superficial and deep-seated, of the spinal chord generally.—The anterior roots are composed of numerous nervous fibres, having nearly the same thickness (that of a hair) each of which arises separately—approaches the neighbouring fibres of the same root, but does not blend with them. The anterior roots, in man at least, do not enter the spinal ganglia. The posterior roots are composed of fibres arising in a triple order, the chief part coming directly from the posterior horns of the grey substance, while other filaments, few in number, take their origin from the white substance of the posterior fasciculi—and others again from the lateral fasciculi.

In comparing the anterior and posterior roots, four circumstances may be remarked, in which they differ. 1st. The filaments of the posterior roots are, generally speaking, thicker and less numerous than the filaments of the anterior roots.—2dly. The filaments of the posterior alone present the plexiform structure. 3dly. The posterior roots alone form the spinal ganglia. 4th. The neighbouring posterior roots almost all communicate by nervous filaments.

In regard to the functions of the parts, M. Bellingeri considers those filaments which come from the anterior fasciculi as serving the purpose of voluntary motion, because they arise from prolongations of the cerebrum;—and he holds that anatomy, physiology, and pathology combine to prove that the brain proper presides over voluntary motion—thus differing in opinion from his colleague Rolando, from Fleurens, and from other recent experimenters. The grounds assigned for his dissent are as follows:—The third pair, or motores oculorum, which are nerves of motion only, rise chiefly from the crura cerebri—and to the sixth, or abductores, nerves purely of motion, arise from the superior extremity of the corpora pyramidalia, productions of the cerebrum. The principal fibres of the twelfth nerve, or hypoglossus, presiding over the motions of the tongue, arise from the external sides of the corpora pyramidalia. He thinks that pathology confirms these anatomico-physiological views of the brain proper being the regulator of voluntary motion. Paralysis, he observes, occurring on the side opposite to the injury received, proves the cerebrum to be the seat of motion; because if hemiplegia occurred from injury of the cerebellum, it could not be on the opposite side, from the absence of any decussations of the prolongations.

In respect to the uses of the posterior roots of the spinal nerves, M. Bellingeri comes to the conclusion, that those filaments coming from the posterior fasciculi are destined for voluntary motion—while those coming from the lateral fasciculi, are supposed to answer the same purposes as the corresponding filaments of the anterior roots—viz. the regulation of the organic functions. The filaments arising from the posterior cornua of the grey matter are supposed to communicate the sense of touch. The posterior roots alone present a pluxuose structure in their origin and course. They constitute the spinal ganglia, and are composed of thicker filaments than the anterior roots. The olfactory nerves, the optic, and the auditory, arise from grey matter, or communicate with it at their origin, and have a ganglioniform structure. On the other hand, the nerves of voluntary motion arise from the white medullary matter—have no pluxuose or ganglioniform structure—and are thinner and finer than the sentient nerves. It is hardly necessary for us to observe, that the physiological portion of M. Bellingeri's remarks are little better than speculations, and very far from being confirmed by unequivocal facts. But the most extravagant of these speculations are yet to come. He declares his belief, that the nerves coming from the cerebrum and its productions, serve for the purpose of flexion and abduction, while those from the cerebellum and its productions, serve for extension and adduction. We conceive that this is downright raving. Is the *action* of a muscle that extends a limb different in kind from that which bends it. Certainly not. M. Bellingeri might just as well conclude, that the cerebrum was for the fingers, and cerebellum for the toes, as that one was destined for muscles that extend, and the other for muscles that bend the body or any of its members.

---

5. *Effect of Ptyalism.** By a process of reasoning into which our limits will not permit us to enter, Mr. Arnott has been led to conclude, that the well known effect of mercury in arresting acute diseases, as hepatitis and dysentery, is owing principally, if not solely, to the counter-irritation produced in the mouth by complete ptyalism. He considers that doctrine erroneous, or at all events, overstrained, which attributes the cure of acute diseases to the general action of mercury on the whole vascular and secretory systems, and the consequent depletion which ensues. Neither does he count for any thing the specific action, supposed by many to be set up in the system, and to supersede other morbid actions going on previously. The *facts* on which the present doctrine of counter-irritation turns are as follow:—On a voyage from India, many of the ship's crew were affected with dysentery. "After trying most of the other remedies that prove serviceable in such cases," says Mr. A. "recourse was obliged to be had to complete mercurial ptyalism before the disease would yield, and in several instances no other practice was followed. In the last five cases that occurred, I tried the effect of powerfully irritating the mouth with powdered capsicum diffused in water, for which its recently prepared tincture was afterwards substituted, as possessing greater strength." The patients put a tea-spoonful of the medicine into the mouth, every five or ten minutes, as the irritation ceased, and retained it until the mouth became filled with saliva. This process was continued with little intermission during the day, or as long as was requisite to afford relief. Great heat and smarting were produced in the mouth, with vesication, at different points of its lining membrane, and the saliva was copiously secreted. The effects were these. "The colicky pains were soon relieved. The dejections soon decreased in frequency; and in one instance, after the employment of the irritant for about twelve hours, there occurred the sudden change in the bowels from irritability to such a torpid state, that, for two days together, there was but one scanty evacuation." In another instance the patient, who applied in the morning for relief from an irritation that appeared to exist both in the mucous membrane of the respiratory and digestive organs, recovered so completely in about twelve hours' application of the capsicum, as to be able to resume his occupation on board, free of complaint, on the third day. "In one case the patient had already taken a large dose of calomel, in about six hours after commencing the use of the irritating tincture, and twelve hours after the exhibition of the calomel, his breath had the mercurial fetor, and he was soon affected with severe mercurial ptyalism. This was the more remarkable, as when the

* Mr. Arnott's Enquiry, &c. See Bibliography.

same man complained of dysentery in a former part of the voyage, it was with difficulty that ptyalism could be produced."

The above are the main facts which are adduced by our author, in support of his theory of local irritation superseding or arresting a general disease. "The above trials," Mr. A. adds, "are, at least, sufficient to shew that powerful irritation of the mouth is adequate to the removal of an inflammatory disturbance of the intestines, and consequently, that the same measure should be of advantage in other analogous diseases."

We are unwilling, by any observation of ours, to weaken the facts brought forward by Mr. Arnott. But we cannot help thinking, that he attaches more importance to the ptyalism—that is, the irritation of the mouth—than it deserves. From what we have seen in others, and felt in ourselves, we should be inclined to consider the mere affection of the salivary glands, as acting a subordinate part in the removal of a severe affection, as of dysentery, for instance. What induces us to dissent from Mr. Arnott, is the repeated observation of dysentery and other diseases abating *before* there was any considerable pain or irritation of the mouth. Every one who has suffered ptyalism, must recollect that the first swelling of the gums and rush of saliva to the mouth are productive of very little inconvenience; though it is at this precise period and sometimes before, that the dysenteric symptoms abate. It is after the ptyalism is a day or two established, and especially when ulceration takes place in the gums, cheeks, and tongue, that the penance begins to be felt. We repeat it, that the first day or two of ptyalism is accompanied by very little irritation, comparatively, and consequently that we do not think the subsidence of the dysenteric affection is owing to counter-irritation in the mouth.

